# Physiological and Molecular Characterization of Allyl Isothiocyanate Adaptation in the Formosan Subterranean Termite, *Coptotermes formosanus*

**DOI:** 10.3390/insects17101088

**Published:** 2026-10-20

**Authors:** Wanyan Jiang, Lijun Cai, Fang Zhu, Timothy W. Moural, Tianyi Yang, Jiafu Zhang, Qi Zhao, Meixiang Wu

**Affiliations:** 1 State Key Laboratory of Agricultural and Forestry Biosecurity, College of Plant Protection, Fujian Agriculture and Forestry University, Fuzhou 350002, China; 2 Biological Control Research Institute, Fujian Agriculture and Forestry University, Fuzhou 350002, China; 3 Institute of Applied Ecology, Fujian Agriculture and Forestry University, Fuzhou 350002, China; 4 Department of Entomology, Penn State University, University Park, PA 16802, USA; fuz59@psu.edu (F.Z.); twm78@psu.edu (T.W.M.); 5 Fuzhou Greening Management Office, Fuzhou 350003, China

**Keywords:** *Coptotermes formosanus*, allyl isothiocyanate, antioxidant enzymes, detoxification enzymes, glutathione S-transferase, molecular docking

## Abstract

The Formosan subterranean termite, *Coptotermes formosanus* is a globally distributed species that feeds on lignocellulose and causes substantial economic losses annually. Currently control strategy heavily relies on chemical pesticides, raising concerns about environmental impacts associated with their extensive use. Allyl isothiocyanate (AITC), a plant-derived compound, has demonstrated potent insecticidal activity against insect pests. However, its physiological and molecular effects in *C. formosanus* remain poorly understood. In this study, transcriptome data were used to identify antioxidant, detoxification, and neural-related genes, followed by phylogenetic analysis. *C. formosanus* was treated with AITC at LC_50_ for 24 h, after which enzyme activities and gene expression profiles were assessed. AITC exposure significantly increased the activities of glutathione S-transferase (GST), carboxylesterase (CarE), acetylcholinesterase (AChE), catalase (CAT), superoxide dismutase (SOD), and peroxidase (POD), accompanied by differential regulation of corresponding gene families. POD genes showed coordinated transcriptional and enzymatic induction, whereas SOD genes displayed isoform-specific responses with strong induction of mitochondrial *CfSOD2* homologs. The detoxification-related genes *CfGSTS1* and *CfGSTO1* were significantly upregulated. Molecular docking and dynamics simulations further indicated dynamic interaction between AITC and CfGSTS1, providing structural evidence for a potential interaction that may be relevant to AITC detoxification. Together, these findings advance our understanding of the physiological and molecular responses of *C. formosanus* to AITC exposure and provide a basis for further evaluation of AITC as a potential alternative termite control agent.

## Simple Summary

The Formosan subterranean termite, *Coptotermes formosanus*, is one of the most destructive termite species worldwide, causing extensive damage to wooden structures and other cellulose-based materials. Although chemical pesticides are widely used for termite management, their environmental impacts have encouraged the search for safer alternatives. Allyl isothiocyanate (AITC), a natural compound derived from plants, has shown promise as an insect control agent. In this study, we investigated how termites respond to AITC exposure by examining changes in their antioxidant defenses, detoxification systems, and nervous system. We found that AITC exposure altered these physiological systems, providing new insights into how termites respond to this plant-derived compound. These findings support further research evaluating AITC as a potential tool for more environmentally compatible termite management.

## 1. Introduction

*Coptotermes formosanus* (Blattodea: Heterotermitidae) is a eusocial insect species with a remarkable capacity for efficient lignocellulose degradation and utilization [1,2]. It exhibits strong dispersal ability and environmental adaptability, causing severe damage to wooden structures and resulting in substantial economic losses annually [3]. Moreover, its potential distribution is projected to expand under ongoing climate change [4]. Current termite control strategies rely heavily on chemical pesticides, including pyrethroids, neonicotinoids, organofluorines, and insect growth regulators [5]. However, the widespread application of these chemicals in buildings and soil raises concerns about environmental contamination and risks to human health.

In recent years, botanical insecticides have attracted increasing attention due to their biodegradability, diverse modes of action, and lower potential for resistance development [6]. Numerous plant-derived compounds, particularly essential oils and their constituents, exhibit strong insecticidal and/or repellent activities against termite species. For example, essential oils from *Cunninghamia konishii* and *Cryptomeria japonica*, as well as their major components, have demonstrated significant toxicity to *C. formosanus* [7,8]. Similarly, 1,8-cineole derived from *Eucalyptus camaldulensis* shows fumigant toxicity and inhibits acetylcholinesterase (AChE) activity, suggesting neurotoxic effects [9]. In addition to essential oils, isothiocyanates represent another important class of plant-derived bioactive compounds. Allyl isothiocyanate (AITC), an organosulfur compound generated through myrosinase-catalyzed hydrolysis of glucosinolates following tissue damage in Brassicaceae plants, has demonstrated potent insecticidal activity against various insect pests [10,11,12,13,14,15]. Previous studies have shown that AITC can alter detoxification and antioxidant enzyme activities and may affect key metabolic targets, including cytochrome c oxidase [16]. However, its effects on specific antioxidant and detoxification enzymes and AChE at the levels of enzyme activity and target-gene expression remain poorly characterized in termites.

In insects, antioxidant enzymes including superoxide dismutase (SOD), catalase (CAT), and peroxidase (POD) play essential roles in mitigating oxidative stress by scavenging reactive oxygen species generated under environmental and chemical challenges. These enzymes are critical for maintaining cellular homeostasis and protecting tissues from oxidative damage [17,18,19]. In parallel, detoxification enzymes such as cytochrome P450s, carboxylesterases (CarEs) and glutathione S-transferases (GSTs) are involved in the metabolism and elimination of xenobiotic compounds, including plant-derived allelochemicals and synthetic insecticides, thereby contributing to insect adaptation to host plants and tolerance to toxic exposure [20,21,22,23]. AChE terminates cholinergic neurotransmission by hydrolyzing acetylcholine (ACh), thus restoring synaptic function. As a member of the clade J esterases, AChE plays a central role in neural transmission and is a well-established target of insecticides that disrupt nervous system activity [22,24]. Given the central roles of antioxidant enzymes, detoxification systems, and AChE in maintaining physiological homeostasis and mediating responses to toxic exposure, these pathways may be involved in the biological responses induced by AITC.

Our previous study determined the fumigation toxicity of AITC against *C. formosanus*, with an LC_50_ of 435.993 μL/L under laboratory conditions [25]. Building on this work, the present study examined responses to AITC at its previously determined LC_50_ by monitoring mortality from 24 to 120 h and measuring the activities of antioxidant and detoxification enzymes and AChE, together with the expression of their corresponding genes. Phylogenetic analyses were conducted to characterize the evolutionary relationships of the selected proteins, while structural modeling and molecular docking were used to examine their potential interactions with AITC. By integrating toxicological, biochemical, molecular, phylogenetic, and structural analyses, this study characterizes physiological and molecular responses associated with AITC exposure in *C. formosanus* and provides a foundation for future functional validation and evaluation of AITC-based treatments as a potential alternative tactic for termite management.

## 2. Materials and Methods

### 2.1. Insects Collection and Maintenance

A colony of *C. formosanus* was collected from Huazhou City (Guangdong Province, China) (21.66° N, 110.64° E) in February 2024. The colony was subsequently maintained in the laboratory for one week in a plastic box (18 cm × 10 cm × 10 cm) at 28 ± 1 °C, 75 ± 5% relative humidity (RH) and a 0 L:24 D before conducting enzyme activity and gene expression assays. All experiments were conducted in an environmental chamber (PRX-350B, Plant Instrument Co., Ltd., Ningbo, China). Water and filter paper were utilized as food sources.

### 2.2. Exposure of C. formosanus to AITC at LC_50_

Worker termites were allocated by weight to control (CK) and AITC-treated (AT) groups, with three biological replicates per group (C1–C3 and A1–A3, respectively). Each biological replicate contained approximately 200 termites. AITC (99.99% purity) was purchased from Yeyuan Biotechnology Co., Ltd. (Shanghai, China). n-hexane (99.99% purity; Macklin Biochemical Co., Ltd., Shanghai, China) was used as the solvent for AITC dilution and as the control treatment. Termites in the AT groups were exposed to AITC at LC_50_ concentration (435.993 μL/L), whereas termites in the CK groups received an equal volume of n-hexane. A PRX-350B Intelligent Artificial Climate Chamber (Plant Instrument Co., Ltd., Ningbo, China) was used for termite rearing. After 24 h of exposure, surviving termites were immediately frozen in liquid nitrogen and stored at −80 °C until subsequent enzyme activity assays and gene expression analyses. Termite mortalities in control, AITC treatment, and untreated groups were recorded at 24, 48, 72, 96, and 120 h after exposure (Figure S1).

### 2.3. Enzyme Activity Assays

Surviving worker termites from each group (C1, C2, C3, A1, A2, A3) after 24 h treatment were collected for enzyme activity assays. For each replicate, 0.1 g termites were pooled, weighed, and homogenized in 1 mL ice-cold extraction buffer (0.01 mol/L phosphate buffer, pH 7.4) according to the manufacturer’s instructions. The homogenate was centrifuged at 8000× *g* for 10 min at 4 °C, and the supernatant was collected as the crude enzyme extract and kept on ice until analysis. Enzyme activity assay kits for AChE [ZC-S0384], CarE [ZC-S0387], GST [ZC-S0333], CAT [ZC-S0351], POD [ZC-S0352], and SOD [ZC-S0350] were supplied by ZCIBIO Technology Co., Ltd. (Shanghai, China).

Enzyme activities were determined using a SpectraMax 190 multi-mode microplate reader (Molecular Devices Co., Ltd., Shanghai, China) according to the manufacturers’ instructions provided with the corresponding assay kits. The absorbance of each enzyme assay was measured at the following wavelengths, 412 nm for AChE, 450 nm for CarE and SOD, 340 nm for GST, 240 nm for CAT, and 470 nm for POD. Enzyme activity units were defined according to the kit protocols as follows: AChE activity was defined as the amount of enzymes required to catalyze the formation of 1 nmol 5-thio-2-nitrobenzoic acid (TNB) per minute; CarE activity was defined as the amount causing an increase in absorbance of 1.0 per minute; GST activity was defined as the amount of enzyme catalyzing the conjugation of 1 μmol 1-chloro-2,4-dinitrobenzene (CDNB) with glutathione (GSH) per minute; CAT activity was defined as the amount of enzyme catalyzing the degradation of 1 nmol H_2_O_2_ per minute; POD activity was defined as the amount of enzyme causing an absorbance change of 0.005 per minute; and SOD activity was defined as the amount of enzyme causing 50% inhibition of the xanthine oxidase-coupled reaction. Enzyme activities were expressed as nmol/min/g fresh weight for AChE and U/g fresh weight for the other enzymes.

### 2.4. RNA Isolation, cDNA Synthesis, Reference Gene Stability Assessment, and RT-qPCR

After 24 h of AITC or control treatment, surviving *C. formosanus* workers from each group were collected for RNA extraction. For each sample, approximately 0.1 g of termite tissue was ground into a fine powder in liquid nitrogen. Total RNA was extracted using a commercial RNA extraction kit (Herui Biotechnology Co., Ltd., Fuzhou, China) according to the manufacturer’s instructions. RNA concentration was measured using a BioDrop μLite Ultra-low-volume spectrometer (BioDrop Ltd., Cambridge, UK). First-strand cDNA was synthesized using OneStep gDNA Removal and Reverse transcription kit (Herui Biotechnology Co., Ltd., Fuzhou, China) following the manufacturer’s instructions. Reverse transcription was performed in a Thermal Cycler (Mona Biotechnology Co., Ltd., Suzhou, China) using total RNA as the template under the following conditions: 42 °C for 15 min, followed by 85 °C for 10 s. The synthesized cDNA was stored at −20 °C for subsequent RT-qPCR analysis. We predicted coding sequences (CDSs) of the transcriptome data using a homology-based approach [25]. Transcript sequences were first searched against the Swiss-Prot and non-redundant (NR) protein databases using BLASTX (2.13.0) [26], and CDSs were inferred based on the best significant alignments. According to the predicted CDSs and phylogenetic analyses, gene specific primers were designed for AChE, CarE, GST, CAT, POD, SOD genes, and reference genes (Table S1). The expression stability of seven candidate reference genes (*EF1-a*, *GAPDH*, *18S*, *NADH5*, *RPL32*, *RPS18* and *RPL*) was evaluated using the ΔCt method, BestKeeper, NormFinder, and geNorm, with RefFinder used to generate the comprehensive stability ranking [27].

RT-qPCR was performed on a Gentier 96R Automated Medical PCR Analysis System (Tianlong Technology Co., Ltd., Xi’an, China) using HRbio qPCR SYBR Green Master Mix (No ROX) (Herui Biotechnology Co., Ltd., Fuzhou, China) following the manufacturer’s protocol. The amplification program consisted of an initial denaturation at 95 °C for 5 min, followed by 40 cycles of denaturation at 95 °C for 10 s, annealing at 58 °C for 20 s, and extension at 72 °C for 20 s. A melt curve analysis was subsequently performed at 95 °C for 1 min, 60 °C for 15 s, and 98 °C for 5 s. Total reaction volume was 20 µL and consisted of 0.4 µL cDNA, 10 µL RT-qPCR SYBR Green Master Mix, 0.4 µL forward primer, 0.4 µL reverse primer (Table S1), and 8.8 µL ddH_2_O. Relative gene expression levels were calculated using the 2^–∆∆Ct^ method [28]. RT-qPCR was performed using three biological replicates, each with three technical replicates.

### 2.5. Phylogenetic Analysis

Based on the predicted CDSs, sequences corresponding to antioxidant-, detoxification-, and neural-related enzymes, including SOD, POD, GST and AChE, were identified and extracted. The predicted CDSs were translated to amino acid sequences and homologous protein sequences were retrieved from the NCBI database for phylogenetic analysis. The accession numbers and full names of all sequences are provided in Table S2. Phylogenetic tree analysis was performed using MEGA 11 software [29]. Amino acid sequences were aligned, and phylogenetic trees were constructed using the Neighbor-Joining method [30]. The robustness of the tree topology was evaluated using bootstrap analysis with 1000 replicates [31], and bootstrap support values were shown at the corresponding nodes. Evolutionary distances were computed using the p-distance method [32]. All other parameters were set to their default values.

### 2.6. GST Structural Modeling and Molecular Docking with AITC

The model complex of CfGSTS1 with GSH was generated using the Boltz-2 program integrated into ChimeraX [33,34]. Then the generated model was compared through structural alignment with our previously reported LdGSTs2 X-ray crystal structure (PDB:9P6K) from *Leptinotarsa decemlineata* [35]. The CfGSTS1-GSH complex model was prepared for docking using Dock Prep in ChimeraX [36]. Molecular docking with allyl isothiocyanate (AITC: PubChem CID 5917) was done three times using GNINA v1.3.3 [37]. Docking search box was centered on GSH and extended by 8 Å in each direction, exhaustiveness set to 32, with 20 poses generated per run, and CNN rescoring. Three docking simulations were run, and a consensus pose was selected for MD simulations based on pose agreement across runs and placement of the electrophilic carbon of AITC relative to the GSH sulfur. Protonation states of ionizable residues in CfGSTS1 were evaluated at pH 6.5 using PROPKA 3.5.1 and incorporated during molecular dynamics simulation preparation [38,39]. Additionally, CfGSTS1 was parameterized with AmberTools using Amber ff19SB, GSH and AITC with GAFF2/AM1-BCC using Antechamber [40,41,42,43]. The complex was solvated in a truncated octahedral water box, and adjusted to 0.15 M NaCl using tleap. The final system contained 55,814 atoms, with 13,086 waters, 39 Na^+^, and 36 Cl- ions [44]. Simulations were performed with CUDA-accelerated OpenMM 8.3.1 using particle-mesh Ewald electrostatics, constrained hydrogen-containing bonds, and a 2 fs timestep [45,46]. Following restrained minimization, the system was heated from 10 to 300 K over 130 ps, equilibrated under restrained NPT conditions for 500 ps at 300 K and 1 bar, and subjected to a 1.2 ns staged restraint release. Three independent unrestrained 10 ns NPT production simulations were then performed from the equilibrated structure using independent seeds. Coordinates were saved every 10 ps, yielding 1000 frames per replica.

### 2.7. Statistical Analysis

Data on enzyme activities and relative gene expression levels among different groups were analyzed using GraphPad Prism 8.0.2 software (GraphPad Software, USA). Normality of all data was confirmed prior to analysis. Differences between groups were assessed using Student’s *t* tests. Statistical significance was set at *p* < 0.05.

## 3. Results

### 3.1. AITC Exposure at LC_50_ Enhances AChE, Detoxification, and Antioxidant Enzyme Activities in C. formosanus

**Figure 1:**
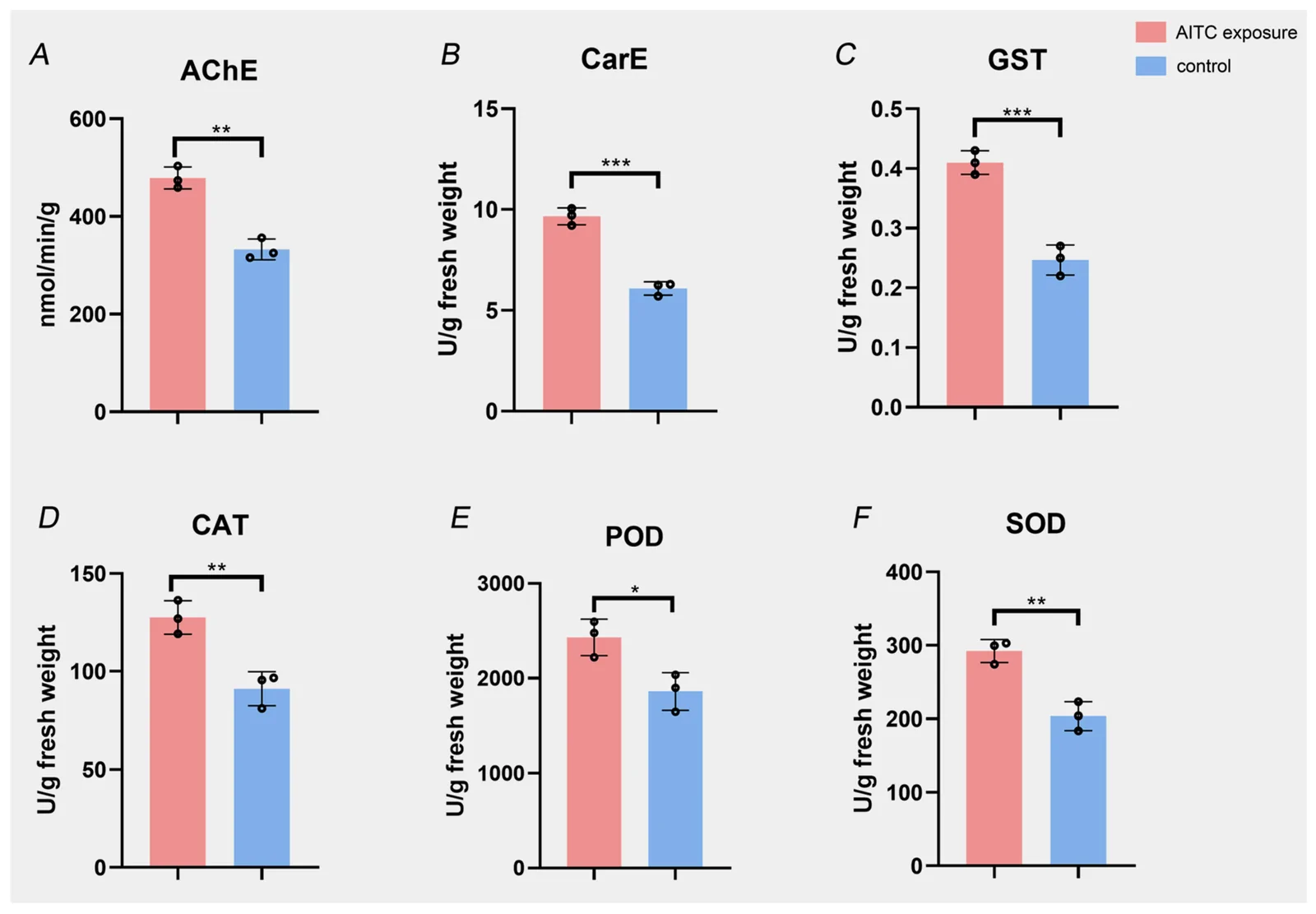
Effects of the LC_50_ of AITC on the activities of (**A**) AChE, (**B**) CarE, (**C**) GST, (**D**) CAT, (**E**) POD, and (**F**) SOD in *C. formosanus.* Enzymatic activities were measured in termites treated with either AITC (red bar) or n-hexane (control, blue bar). Data are presented as the mean ± SD (*n* = 3). Each group has three biological replicates. Statistical significance between the AITC-treated and control groups was determined by Student’s *t* tests. * *p* < 0.05, ** *p* < 0.01 and *** *p* < 0.001 indicate significant differences; NS, not significant.

To elucidate the effects of AITC at LC_50_ on antioxidant, detoxification, and AChE activities in *C. formosanus*, we measured the enzyme activities in termites exposed to n-hexane (control, CK) or AITC at its LC_50_ (435.993 μL/L). Relative to the control, AITC treatment significantly increased the activities of all enzymes tested, including AChE, CarE, GST, CAT, POD, and SOD. Specifically, the activity of neural-related enzyme AChE increased by 44% relative to the control group. Among the detoxification enzymes, the activities of CarE and GST elevated by 59% and 64%, respectively (Figure 1). The activities of antioxidant enzymes CAT, POD, and SOD also increased by 40%, 31%, and 43%, respectively, compared with the control group (Figure 1). In addition, the mortality in CK (n-hexane) and untreated groups showed no significant difference (Figure S1).

### 3.2. AITC Exposure at LC_50_ Induces Differential Gene Expression in C. formosanus

**Figure 2:**
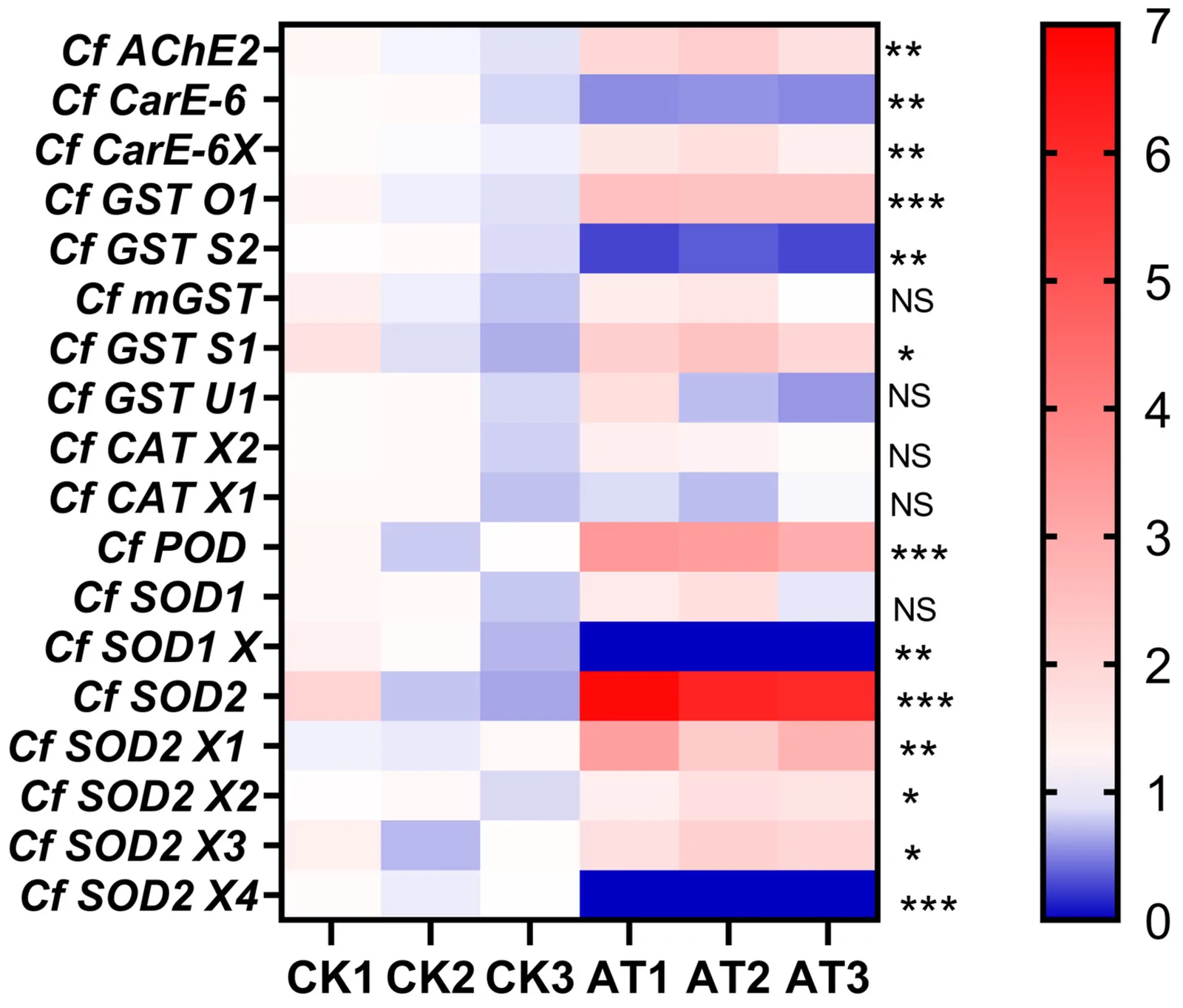
Effects of AITC at LC_50_ on the relative expression of antioxidant-, detoxification-, and neural-related genes in *Coptotermes formosanus.* Relative transcript levels were determined by RT-qPCR in termites exposed to AITC or n-hexane. Three biological replicates were performed, and each containing three technical replicates. Relative express levels are shown, with higher values in red and lower values in blue. Statistical significance between the AITC-treated and control groups was determined using Student’s *t* tests. * *p* < 0.05, ** *p* < 0.01 and *** *p* < 0.001 indicate significant differences; NS, not significant.

To select the stable reference genes, we performed RT-qPCR and analyzed Ct-value of each candidates reference genes. *GAPDH* and *NADH5* were selected as reference genes for qPCR normalization (Table S3), and the geometric mean of the two reference genes was used for normalization. We then assessed the effects of AITC exposure on the gene expression in *C. formosanus*, by measuring the relative expression levels of enzyme-encoding genes in termites treated with either n-hexane (control) or an LC_50_ of AITC (Figure 2). Among 18 genes examined, 9 exhibited significant induction, 4 showed significant repression, and the remaining 5 showed no significant changes in expression. Among the upregulated genes, *CfSOD2* showed the strongest induction, with its relative expression level reaching 5.5-fold that of the control group. The next highest induction levels were observed for *CfGSTO1* (3.17-fold) and *CfSOD2X1* (2.27-fold) (Figure 2).

At the gene family level, the expression of *CfAChE2* and *CfPOD* was significantly increased following AITC exposure. In contrast, the two *CarE* genes exhibited opposite expression patterns: *CfCarE-6X* was significantly upregulated, whereas *CfCarE-6* was significantly downregulated (Figure 2). The GST family also exhibited differential responses. Among the five *GST* genes examined, only *CfGSTO1* and *CfGSTS1* were significantly induced, whereas *CfGSTS2* was significantly downregulated to approximately 30% of the control expression level (Figure 2). The SOD family showed isoform-specific responses to AITC. Among the seven *SOD* genes examined, *CfSOD2*, *CfSOD2X1*, *CfSOD2X2*, and *CfSOD2X3* were significantly up-regulated, with *CfSOD2* and *CfSOD2X1* showing the two highest induction levels among all genes analyzed. In contrast, *CfSOD2X4* and *CfSOD1X* was significantly downregulated, whereas the remaining one *SOD* genes showed no significant changes in expression. By comparison, neither of the two *CAT* genes (*CfCATX1*, *CfCATX2*) exhibited significant transcriptional changes following AITC exposure (Figure 2).

### 3.3. Phylogenetic Relationships of Key Enzymes in C. formosanus and Other Insects

**Figure 3:**
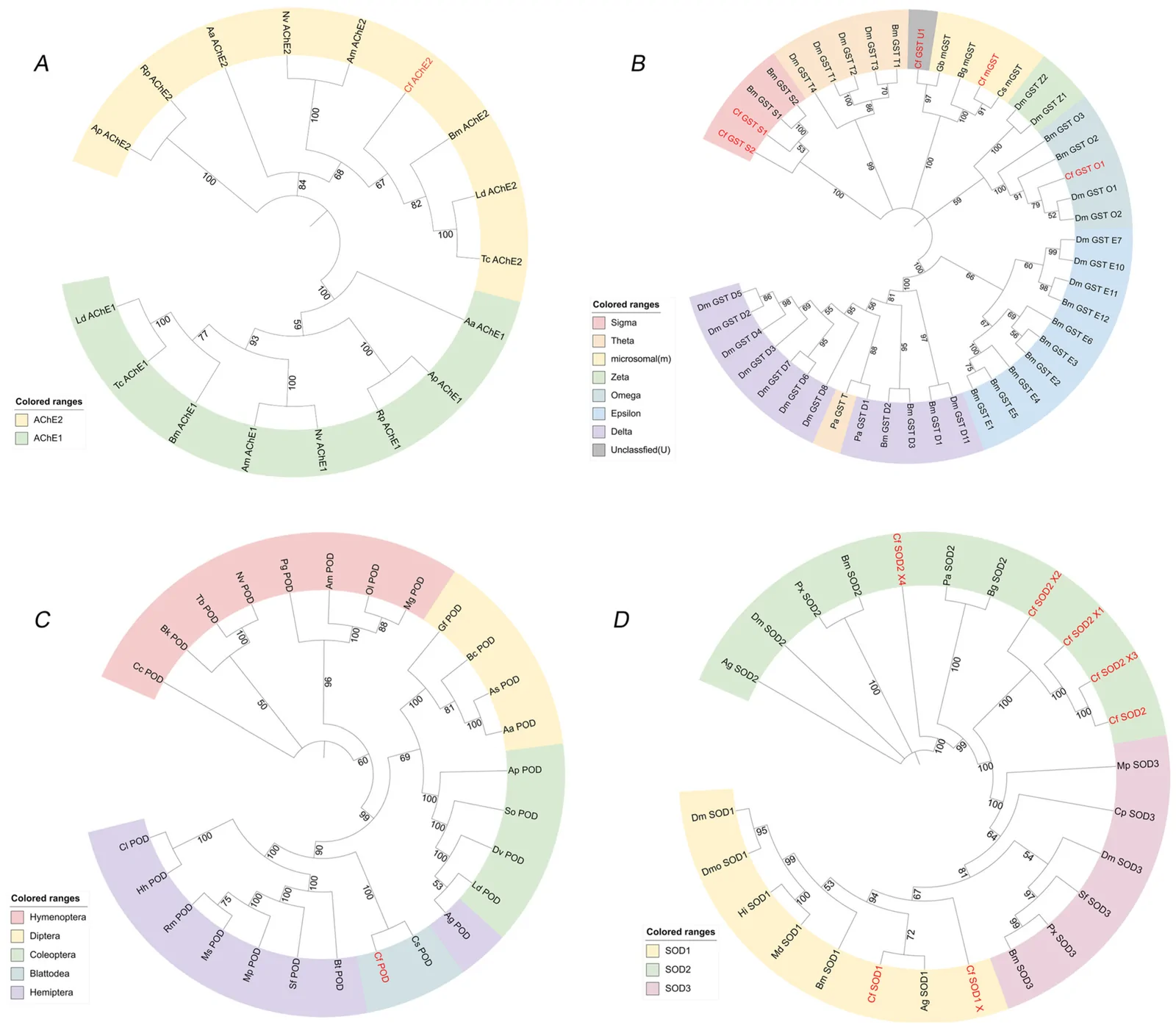
Phylogenetic relationships of selected enzyme families in *C. formosanus* and other insect species. Phylogenetic trees were constructed for four gene families in *C. formosanus*: (**A**) AChE, (**B**) GST, (**C**) POD, and (**D**) SOD. Predicted amino acid sequences of *C. formosanus* (highlighted in red) were aligned with homologous sequences from other insects (e.g., *Cryptotermes secundus*, *Zootermopsis nevadensis*).

A total of 18 genes encoding target enzymes examined in this study were identified from predicted coding sequences in the *C. formosanus* transcriptome [25]. Conserved-domain searches and NCBI BLAST (2.13.0) analyses were used for initial sequence annotation. Phylogenetic analysis of AChE, GST, POD, and SOD families placed the *C. formosanus* sequences relative to homologs from other insects, including *Cryptotermes secundus*, *Zootermopsis nevadensis* (Table S2; Figure 3A–D). Based on the combined domain, sequence-similarity, and phylogenetic evidence, consistent gene names were assigned for use throughout this study.

Insect AChEs formed two major clades, AChE1 and AChE2, with *CfAChE2* clustering within the AChE2 clade (Figure 3A). Multiple sequence alignment further supports this assignment (Figure S2). The five *CfGST* sequences were distributed among the Omega, Sigma, Microsomal, and unclassified GST groups (Figure 3B). POD homologs generally grouped according to insect order, with *CfPOD* and *CrsPOD* from *Cryptotermes secundus* forming a well-supported Blattodea clade (Figure 3C). The *CfSOD* sequences were assigned to the SOD1 and SOD2 subfamilies (Figure 3D).

### 3.4. Structural Modeling of CfGSTS1 and Molecular Docking Analysis of AITC

**Figure 4:**
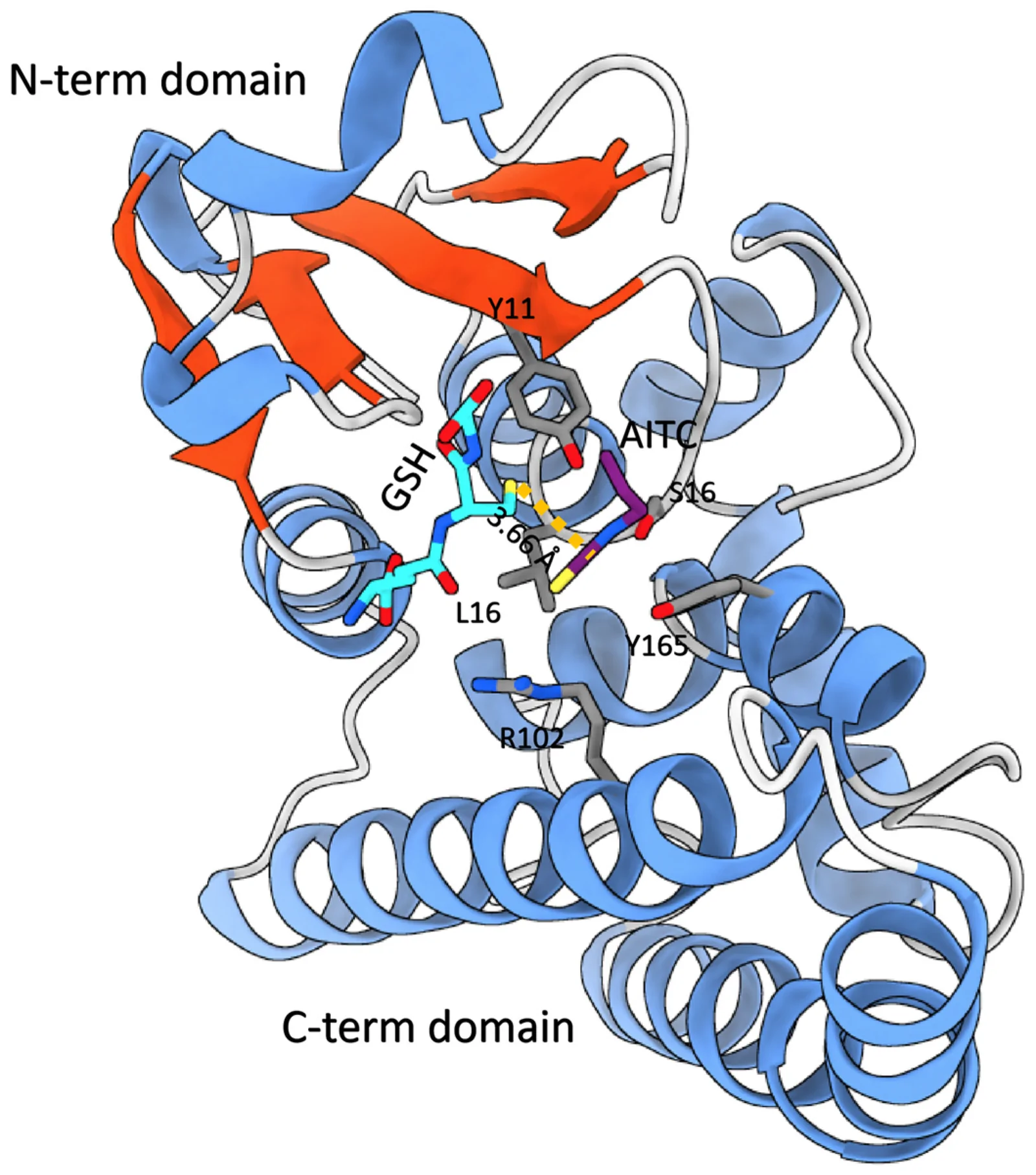
AITC docked pose in the CfGSTS1 active site. The structure shown is from GNINA docking pose with highest rank following explicit-solvent minimization, heating, restrained NPT equilibration, staged restraint release, and 400 ps of unrestrained molecular dynamics using the protein protonation state assigned for pH 6.5. The GST is shown as a cartoon, with α-helices in light blue, β-strands in orange, and coils in light gray. The N-and C-terminal domains are indicated. GSH and allyl isothiocyanate (AITC) are shown as sticks with cyan and purple carbon atoms, respectively. CfGSTS1 residues within 4 Å of AITC are shown as sticks and labeled (Tyr11, Ser16, Leu17, Arg102, and Tyr165). The yellow dashed line indicates the 3.66 Å distance between the GSH sulfur and the electrophilic carbon of AITC. Heteroatoms are colored by elements.

The CfGSTS1-GSH complex model generated with Boltz2 had an overall confidence score of 0.9656, pTM of 0.9629, iPTM of 0.9656, complex pLDDT of 0.9643, and ligand ipTM of 0.9656 (Figure 4). The docked AITC molecule was positioned adjacent to GSH within the predicted CfGSTS1 active site within the H-site. In the AITC pose, the electrophilic carbon was positioned 3.6 Å from the SG2 sulfur atom of GSH and the binding pocket of the AITC molecule was formed by Tyr11, Ser16, Leu17, Arg102 and Tyr165 (Figure 4). The three 10 ns MD simulations resulted varied positioning of AITC, with the electrophilic carbon of AITC mean distance from the SG2 GSH atom were 1.399 ± 9.600 Å, 4.986 ± 1.852 Å and 6.161 ± 1.664 Å for the three replicate runs. Across simulations, starting pocket contact was observed in 72.2% of frames, the GSH cysteinyl sulfur was at ≤4.0 Å distance from the electrophilic carbon of AITC in 27.0% of frames, and near attack geometries (70–110°) occurred in 22.6% of frames. Within the simulated timescale, AITC did not remain stably associated in a ternary CfGSTS1–GSH–AITC complex but transiently sampled positions proximal to GSH within the predicted CfGSTS1 active site. Together, the CfGSTS1–GSH model, docking results, and MD simulations suggest a potential dynamic interaction between CfGSTS1 and AITC and provide a structural hypothesis for future biochemical validation.

## 4. Discussion

Under adverse stress conditions, insects activate physiological defense mechanisms, including detoxification and antioxidant pathways, to maintain cellular homeostasis [17,18]. Among these, CAT, POD, and SOD, constitute the primary protective enzyme systems, functioning coordinately to regulate reactive oxygen species (ROS) and mitigate oxidative damage. SOD catalyzes the dismutation of superoxide anion radicals to generate hydrogen peroxide (H_2_O_2_), which is subsequently decomposed into water and oxygen by CAT and POD, thus maintaining cellular redox balance [47,48,49]. In the present study, AITC exposure significantly altered the activities of antioxidant and detoxification enzymes in *C. formosanus*, indicating the activation of these defense mechanisms in response to AITC-induced stress. Similar responses have been reported in other insects under insecticide stress. For instance, in *Trichogramma ostriniae*, significant increases in the activities of SOD, POD, and CAT were observed after exposure to beta-cypermethrin [50].

AChE plays a critical role in cholinergic synaptic transmission by hydrolyzing ACh, terminating nerve impulses and maintaining normal neural functions [24]. In current study, AITC exposure significantly elevated AChE activity and upregulated *CfAChE2* gene expression. This response may reflect a compensatory or adaptive mechanism to maintain neural homeostasis under chemical stress, rather than direct inhibition of AChE activity. Unlike classical neurotoxic insecticides that inhibit AChE [51,52], the observed increase in AChE activity suggests that AITC may not act as a typical AChE inhibitor. Similar increases in AChE activity have been reported in *Nilaparvata lugens* following exposure to chlorantraniliprole and imidacloprid [53]. Interestingly, although total CAT activity was significantly elevated following AITC exposure, neither of the two *CAT* genes exhibited differential expression between the control and AITC-treated termites (Figure 1 and Figure 2). Discordance between catalase transcript and protein abundance has also been reported in *Drosophila melanogaster*, supporting regulation beyond transcription [54]. CAT activity can be altered through post-translational modification, including phosphorylation-dependent changes in enzyme assembly and activity [55], while ubiquitination and proteasomal degradation can regulate CAT stability [56]. Redox-sensitive peroxisomal import may additionally influence CAT localization and cellular function [57]. Thus, the observed activity–transcript mismatch may reflect temporal decoupling, post-transcriptional or post-translational regulation, or altered protein turnover. These possibilities remain hypothetical and require time-resolved measurements of CAT transcripts, protein abundance, modification state, and subcellular localization. Similar inconsistencies between antioxidant enzyme activity and gene expression have been reported in insects exposed to oxidative stressors, highlighting the complexity of antioxidant defense regulation [17]. PODs are important antioxidant enzymes that contribute to ROS detoxification by reducing H_2_O_2_ and maintaining cellular redox homeostasis [17,58]. Here, we observed that AITC exposure significantly increased both POD activity and *POD* transcript expression, suggesting that the POD-mediated antioxidant defense pathway represents a major inducible component in response to AITC-induced oxidative stress.

SODs play a central role in the first line of enzymatic defense against oxidative stress by catalyzing the dismutation of superoxide radicals (O_2_•^−^) into H_2_O_2_, preventing excessive ROS accumulation [17,59]. In insects, SODs are generally classified into cytosolic Cu/Zn-SOD (SOD1), mitochondrial Mn-SOD (SOD2), and extracellular Cu/Zn-SOD (SOD3) based on their cellular localization [59]. Our study revealed that the *SOD* genes identified in *C. formosanus* belong to SOD1 and SOD2 subfamilies, with no SOD3 homologs detected in the current transcriptome (Figure S3). Four putative *SOD* paralogs formed a species-specific cluster, suggesting a lineage-specific expansion (Figure 3D). If confirmed at the genomic level, this expansion could increase antioxidant capacity through a gene-dosage effect or allow regulatory and functional divergence among paralogs, including tissue-, developmental stage-, or stress-specific expression. Previous studies have shown that AITC can impair mitochondrial function and increase mitochondrial ROS production in eukaryotic cells [60]. Consistent with this observation, the two *SOD1* homologs in *C. formosanus* exhibited distinct transcriptional responses to AITC exposure, with one showing no significant change and the other being significantly downregulated. In contrast, four *SOD2* genes were markedly upregulated (Figure 2). This coordinated expression pattern is consistent with an enhanced transcriptional response of putative SOD2-family genes to AITC-associated oxidative stress [60], but it does not by itself demonstrate preferential activation of mitochondrial antioxidant defenses. Expression divergence among paralogs may arise from differences in *cis*-regulatory architecture, while epigenetic regulation of SOD2 has also been reported in other eukaryotes [61,62]. Moreover, SOD2 activity can be influenced by mitochondrial import, manganese incorporation, and post-translational acetylation or deacetylation [63,64]. In *Chilo suppressalis*, *MnSOD* (SOD2) expression was significantly upregulated following exposure to chlorpyrifos, abamectin, and chlorantraniliprole, further supporting the role of mitochondrial SOD2 in insect responses to xenobiotic stress [65]. Similar differential regulation of SOD paralogs under oxidative stress has also been reported in *Bombyx mori* under rotenone-induced mitochondrial stress [66]. The increased total SOD activity, together with the induction of multiple *SOD2* genes, suggests that mitochondrial SOD2 contributes to the enhanced antioxidant capacity following AITC exposure.

GSTs are major detoxification enzymes that catalyze the conjugation of electrophilic xenobiotics with glutathione and contribute to cellular protection against oxidative stress by regulating reactive electrophile and ROS levels [67]. Among the GST subclasses, Omega class GSTs are particularly associated with redox regulation and antioxidant defense through their roles in maintaining cellular redox homeostasis and metabolizing reactive electrophilic compounds. In contrast, Sigma class GSTs are primarily recognized for their roles in xenobiotic detoxification but also contribute to antioxidant protection by mitigating oxidative damage [23]. In the present study, total GST activity was significantly elevated following AITC exposure, and two of the five GST genes identified in the *C. formosanus* transcriptome, including one Omega-class GST and one Sigma-class GST, were significantly induced. Furthermore, molecular docking analysis predicted that AITC could bind to the H-sites of CfGSTS1 protein, suggesting its potential capacity for AITC recognition. Consistent with the docking results, MD simulations indicated that AITC could transiently occupy a pre-reactive position adjacent to GSH within the CfGSTS1 active site, indicating that CfGSTS1 may interact with AITC and potentially contribute to its detoxification. Several studies have reported the involvement of GSTs in isothiocyanate (ITC) detoxification across a range of herbivorous insects. In generalist lepidopterans, including *Spodoptera littoralis*, glucosinolate-derived isothiocyanates from Brassicaceae plants are metabolized into glutathione conjugate derivatives, indicating that GST-mediated conjugation represents an important pathway for the detoxification of these electrophilic compounds [68]. In *D. melanogaster*, GSTD2 catalyzes the glutathione conjugation of isothiocyanates, whereas GSTE7 efficiently metabolizes several ITCs and enhances tolerance to dietary ITCs when overexpressed [69,70]. In addition, mustard-feeding *Scaptomyza* species utilize a GST-dependent mercapturic acid pathway to detoxify mustard oils, highlighting the contribution of conserved GST-mediated detoxification systems to insect adaptation to plant chemical defenses [71]. Given that AITC is an electrophilic isothiocyanate, the increased GST activity, transcriptional induction of *CfGSTS1* and *CfGSTO1*, and computationally predicted transient positioning of AITC near GSH within the CfGSTS1 H-site are consistent with the hypothesis that GSTs may contribute to the response of *C. formosanus* to AITC. However, these findings do not demonstrate that CfGSTS1 directly binds or catalyzes the conjugation of AITC, or that its induction confers protection. Future work combining in vitro enzymatic assays, identification of AITC–glutathione conjugates, and structural approaches such as X-ray crystallography will be required to test this hypothesis and determine whether CfGSTS1 contributes to AITC metabolism.

In summary, this study integrates physiological, biochemical, and transcriptional analyses of the response of *C. formosanus* to AITC with phylogenetic and structural characterization of selected proteins. The results show that AITC exposure is associated with changes in antioxidant and detoxification systems, providing a foundation for future studies to determine their functional contributions to AITC tolerance and toxicity. Future studies could use RNA interference or CRISPR/cas 9 to test the roles of candidate genes together with biochemical or biophysical assays to validate interactions between AITC and candidate proteins. Experiments incorporating multiple AITC concentrations and time-course analyses of molecular responses, including comparisons between sublethal and lethal exposure conditions, and measurements of feeding inhibition, behavioral changes, and energy metabolism, would help distinguish responses directly contributing to toxicity from compensatory changes or secondary consequences of general stress. Combined with functional validation, these approaches would provide stronger evidence for causal and mechanistic interpretations.

## Data Availability

Data will be made available on request.

## Supplementary Materials

The following supporting information can be downloaded at https://www.mdpi.com/article/10.3390/insects17101088/s1, Figure S1: Fumigation bioassay setup and mortality of worker termites after AITC exposure, n-hexane treatment and untreated control. Figure S2: Alignment of the deduced amino acid sequences encoded by CfAChE2 *Coptotermes formosanus* and orthologs from other insect species including *Apis mellifera ligustica*, *Aedes albopictus*, and *Rhopalosiphum padi*. The Y/D and M/Y typing sites distinguishing insect AChE1 and AChE2 are indicated with blue arrowheads. The AChE signature motif is boxed. Sequence alignment was visualized using ESPript 3.2. Figure S3: Alignment of the deduced amino acid sequences encoded by CfSOD2 and its four variants (CfSOD2X1, CfSOD2X2, CfSOD2X3, CfSOD2X4) from *Coptotermes formosanus* and orthologs from other insect species including *Bombyx mori* (NP_001037299.1), EHJ68139.1 (*Danaus plexippus*), ABR08302.1 (*Galleria mellonella*), and AIJ00868.1 (*Plutella xylostella*). Four conserved manganese-binding residues are indicated with blue arrowheads. The MnSOD signature motif is boxed. Sequence alignment was visualized using ESPript 3.2. Table S1: Primers used in quantitative reverse transcription PCR (RT-qPCR). Table S2: Sequences used for phylogenetic tree construction. Table S3: Stability of candidate reference genes under control and AITC-treated *Coptotermes formosanus*, evaluated using different methods.
