# Supplementary material for: Physiological and Molecular Characterization of Allyl Isothiocyanate Adaptation in the Formosan Subterranean Termite, Coptotermes formosanus
Source: Insects. 2026 Oct 20;17(10):1088. doi: 10.3390/insects17101088 (PMC13704987; doi:10.3390/insects17101088)
Supplement: Supplementary file 1 [file insects-17-01088-s001.zip › insects-4526749-supplementary.pdf]

insects

MDPI

Supplementary Materials

Physiological and Molecular Characterization of Allyl Isothiocyanate Adaptationin the Formosan subterranean termite, *Coptotermes formosanus*

Wanyan Jiang [1, 2], Lijun Cai [1, 3], Fang Zhu [4], Timothy W. Moural [4], Tianyi Yang [1], Jiafu Zhang [1], Qi Zhao [5], and Meixiang Wu *, [1,2]

Table S1. Primers used in quantitative reverse transcription PCR (RT-qPCR).

Gene names	Forward Primer (5′-3′)	Reverse Primer (5′-3′)
CfAChE2	GGGAAGCCTATGCCCAATGA	GCTGTAGTTCGTGGACCCTT
CfCarE-6	ACTGACCCTGATGAAACCCA	GCAGTTGGTTCCCAGACGAA
CfCarE-6X	CTCCGATATGTGTCCAGCGA	CTCTAGCCGGTATCCTTGGC
CfGSTO1	TGCCAGGGATGCTGGATTAC	CCTTCATTGCTTCTTTCCACTCA
CfGSTS2	CTTCACACACGCAGAATGGC	GCGAACGATTCCGGTAAAGC
CfmGST	TGCACATCAGAACACGTTGG	GGAAGACAACACCAGCACCT
CfGSTS1	ACTTCGTTGGCATCCTTGAC	CAGAGCCTTCAGCTTCGGAT
CfGST U1	CCTCCCAGCTTCCCATCAAG	CAGAACTGAATTGCTCCTTCTCA
CfCATX2	ATCTGGGACCTTGTGGGAAAC	CTGCGAGTGGATGAATAAAGGGA
CfCATX1	AAACAGCAACCGCCACTCAA	TAGTTGGCTCCCAGTCGGT
CfPOD	GCCGACATTTGCCTGCATTA	TGCCCAGCTCATACCAGAAC
CfSOD1	TTGGTAAGGGAGGACATGAGC	TGCAATTCCCACCACTCCAC
CfSOD1X	GACCGATGCTGAGGGTAACG	CGTGGATGACGAGAGTACGG
CfSOD2	GAACATCAGACGCCAAGTGC	GGGTCAAGAAGCACCATCCA
CfSOD2X1	GATTACGAGAACCGGAAGGC	TTGATGTTCGCCTCCACGAA
CfSOD2X2	TTTCGGTGGCTTCGACAAGT	ACGGCCTTATCAGCCTTCTG
CfSOD2X3	CTTGGACACATGGGAGCACA	AGTGCCAGTTCACACCAGTC
CfSOD2X4	GGAGCACGCCTACTACCTTC	CTTCTCAACCGCCTTCCAGT
18 S	CGAGATTGAGCAATAACAGGTC	ACGTAATCAACGCGAGCTTATG
GAPDH	TGTTGGGCAGGCAGTAAGAG	GGCCGATACGACCAAATCCA
PRL32	CGCCATCAGAGTGACCGTTA	GACCCTTGAAACGCCTACGA
EF1-α	GTTCGCACATCGACAGCAAG	CAAGGGAATGAGTGACCGCT
PRS18	ATTATGGCAAACCCCCGTCA	CACGCAGTTTGCTGTCCAAG
NADH5	TTCTTGTTGTTTTGGCGGCT	GCAGACACAGGAGTAGGAGC
RPL	ATTGACAACAGAGTTCGTAGGC	CGTTATGCACGAGTACCTTCC

Table S2. Sequences used for phylogenetic tree construction.

Group of Enzyme	NCBI Access Number	Gene Name	Species
	XP_001948653.1	Ap AChE1	*Acyrthosiphon pisum*
	AAT76530.1	Rp AChE1	*Rhopalosiphum padi*
	NP_001280548.1	Tc AChE1	*Tribolium castaneum*
	AEI70751.1	Ld AChE1	*Leptinotarsa decemlineata*
	NP_001037380.1	Bm AChE1	*Bombyx mori*
	XP_393751.1	Am AChE1	*Apis mellifera ligustica*
**AChE**	XP_008214576.1	Nv AChE1	*Nasonia vitripennis*
	BAE71348.1	Aa AChE1	*Aedes albopictus*
	XP_001948988.1	Ap AChE2	*Acyrthosiphon pisum*
	AAU11285.1	Rp AChE2	*Rhopalosiphum padi*
	ADU33190.1	Tc AChE2	*Tribolium castaneum*
	AEI70752.1	Ld AChE2	*Leptinotarsa decemlineata*
	NP_001108113.1	Bm AChE2	*Bombyx mori*

	NP_001035320.1	Am AChE2	*Apis mellifera ligustica*
	XP_001605568.2	Nv AChE2	*Nasonia vitripennis*
	BAE71347.1	Aa AChE2	*Aedes albopictus*
**GST**	NP_001108463.1	Bm GST T1	*Bombyx mori*
	NP_001036994.1	Bm GST S2	*Bombyx mori*
	NP_001036974.1	Bm GST D2	*Bombyx mori*
	NP_001108460.1	Bm GST E4	*Bombyx mori*
	NP_001037197.1	Bm GST E1	*Bombyx mori*
	NP_001037077.1	Bm GST S1	*Bombyx mori*
	NP_001037546.1	Bm GST D3	*Bombyx mori*
	NP_001108464.1	Bm GST E5	*Bombyx mori*
	XP_021205855.1	Bm GST E6	*Bombyx mori*
	NP_001037420.1	Bm GST E2	*Bombyx mori*
	NP_001040435.1	Bm GST O3	*Bombyx mori*
	NP_001037406.1	Bm GST O2	*Bombyx mori*
	NP_001108466.1	Bm GST E3	*Bombyx mori*
	NP_001286857.1	Bm GST E12	*Bombyx mori*
	NP_001034042.1	Bm GST D1	*Bombyx mori*
	NP_524914.3	Dm GST D5	*Drosophila melanogaster*
	NP_788656.1	Dm GST D3	*Drosophila melanogaster*
	NP_525114.1	Dm GST D7	*Drosophila melanogaster*
	NP_524916.1	Dm GST D8	*Drosophila melanogaster*
	NP_524915.1	Dm GST D6	*Drosophila melanogaster*
	NP_524913.1	Dm GST D4	*Drosophila melanogaster*
	NP_524912.1	Dm GST D2	*Drosophila melanogaster*
	NP_001286575.1	Dm GST E11	*Drosophila melanogaster*
	NP_001286570.1	Dm GST E10	*Drosophila melanogaster*
	NP_001285495.1	Dm GST T3	*Drosophila melanogaster*
	NP_724816.3	Dm GST T2	*Drosophila melanogaster*
	NP_572886.2	Dm GST T4	*Drosophila melanogaster*
	NP_650183.1	Dm GST D11	*Drosophila melanogaster*
	NP_610509.2	Dm GST T1	*Drosophila melanogaster*
	NP_729388.1	Dm GST O2	*Drosophila melanogaster*
	NP_648237.1	Dm GST O1	*Drosophila melanogaster*
	NP_611329.1	Dm GST E7	*Drosophila melanogaster*
	NP_649894.1	Dm GST Z1	*Drosophila melanogaster*
	NP_731358.1	Dm GST Z2	*Drosophila melanogaster*
	AEV23869.1	Pa GST T	*Periplaneta americana*
	QGA89197.1	Pa GST D1	*Periplaneta americana*
	XP_023725149.1	Cs microsomal GST	*Cryptotermes secundus*
	PSN47492.1	Bg microsomal GST	*Blattella germanica*
	>GLH04944.1	Gb microsomal GST	*Gryllus bimaculatus*
**POD**	XP_008203489.1	Nv POD	*Nasonia vitripennis*
	XP_014244357.1	Cl POD	*Cimex lectularius*
	XP_014291722.1	Hh POD	*Halyomorpha halys*
	XP_015600699.1	Cc POD	*Cephus cinctus*
	XP_018321308.1	Ap POD	*Agrilus planipennis*
	XP_018907162.1	Bt POD	*Bemisia tabaci*
	XP_020300497.1	Pg POD	*Pseudomyrmex gracilis*
	XP_021705031.1	Aa POD	*Aedes aegypti*
	XP_021920027.1	Zn POD	*Zootermopsis nevadensis*
	XP_022169097.1	Mp POD	*Myzus persicae*
	XP_023023759.1	Ld POD	*Leptinotarsa decemlineata*

	XP_033608583.1	Cs POD	*Cryptotermes secundus*
	XP_025207676.1	Ms POD	*Melanaphis sacchari*
	XP_025418942.1	Sf POD	*Sipha flava*
	XP_016768434.2	Am POD	*Apis mellifera*
	XP_026809437.1	Rm POD	*Rhopalosiphum maidis*
	XP_027843669.1	Ag POD	*Aphis gossypii*
	XP_028147982.1	Dv POD	*Diabrotica virgifera virgifera*
	XP_030749417.1	So POD	*Sitophilus oryzae*
	CAB0040970.1	Tb POD	*Trichogramma brassicae*
	XP_033217653.1	Bk POD	*Belonocnema kinseyi*
	XP_035895474.1	As POD	*Anopheles stephensi*
	XP_037029522.1	Bc POD	*Bradysia coprophila*
	XP_037896461.1	Gf POD	*Glossina fuscipes*
	XP_033333210.2	Mg POD	*Megalopta genalis*
	XP_034181239.2	Ol POD	*Osmia lignaria lignaria*
**SOD**	NP_476735.1	Dm SOD1	*Drosophila melanogaster*
	NP_476925.1	Dm SOD2	*Drosophila melanogaster*
	NP_610682.2	Dm SOD3	*Drosophila melanogaster*
	AIJ00868.1	Px SOD2	*Plutella xylostella*
	AIJ00867.1	Px SOD3	*Plutella xylostella*
	WP_012840828.1	Bg SOD2	*Blattella germanica*
	XP_311594.2	Ag SOD1	*Anopheles gambiae*
	XP_314490.4	Ag SOD2	*Anopheles gambiae*
	WP_012821491.1	Pa SOD2	*Periplaneta americana*
	NP_001037084.1	Bm SOD1	*Bombyx mori*
	NP_001037299.1	Bm SOD2	*Bombyx mori*
	NP_001037358.2	Bm SOD3	*Bombyx mori*
	>UZT55352.1	Sf SOD3	*Spodoptera frugiperda*
	>AXY54994.1	Mp SOD3	*Microdera punctipennis*
	>ALR99790.1	Cp SOD3	*Chrysomela populi*
	>XP_075160361.1	Hi SOD1	*Haematobia irritans*
	>EDW19257.1	Dmo SOD1	*Drosophila mojavensis*
	>NP_001295981.1	Md SOD1	*Musca domestica*

Table S3. Stability of candidate reference genes under control and AITC-treated *Coptotermes formosanus*, evaluated using different methods.

	GAPDH	18S	NADH5	RPL32	RPS18	RPL	EF1-a
CK1-Ct	22.69	23.69	20.83	23.26	26.08	20.47	20.53
CK2-Ct	22.68	19.10	20.77	20.63	25.25	22.03	18.09
CK3-Ct	22.78	18.29	19.98	20.76	23.73	20.64	17.19
AT1-Ct	23.84	17.68	19.52	21.49	23.37	21.79	20.32
AT2-Ct	24.19	18.25	19.35	18.52	23.36	21.83	16.26
AT3-Ct	23.92	17.71	19.46	19.50	23.73	22.49	17.45
Standard Deviation of comparative ΔCt by ΔCt	1.79	2.06	1.31	1.53	1.34	1.81	1.68
Pearson's correlation (r) by BEST Keeper	0.63	1.52	0.53	1.14	0.94	0.66	1.41
Stability Value by NormFinder	1.49	1.83	0.30	1.00	0.44	1.56	1.22
Average of expression stability values by geNorm	0.65	1.65	1.09	1.41	1.20	0.65	1.48
Comprehensive ranking of RefFinder	2	7	1	5	3	4	6

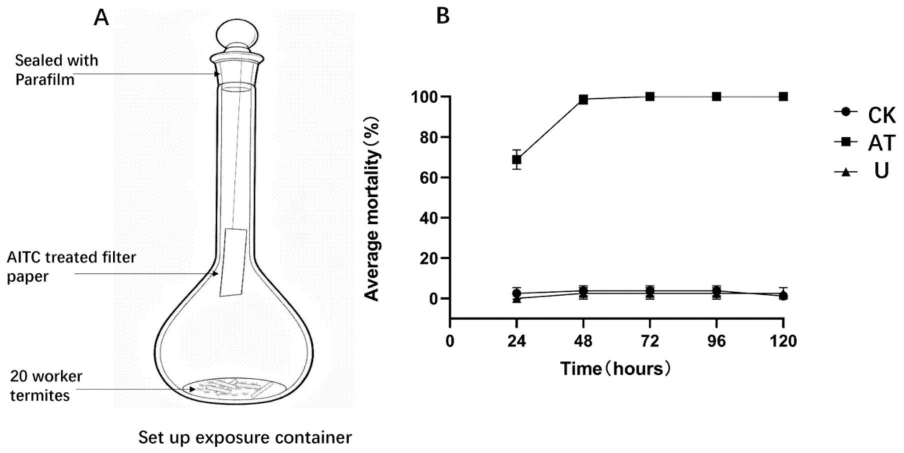

Figure S1. Fumigation bioassay setup and mortality of worker termites after AITC exposure, n-hexane treatment and untreated control. (**A**) Schematic diagram of sealed exposure flask. Twenty worker termites were placed inside in the volumetric flask with filter paper at the bottom serving as food source. (**B**) Average mortality of termites in CK (n-hexane), AT (AITC), and U (untreated) at 24, 48, 72, 96, and 120 hours. Data are presented as mean ± SD.

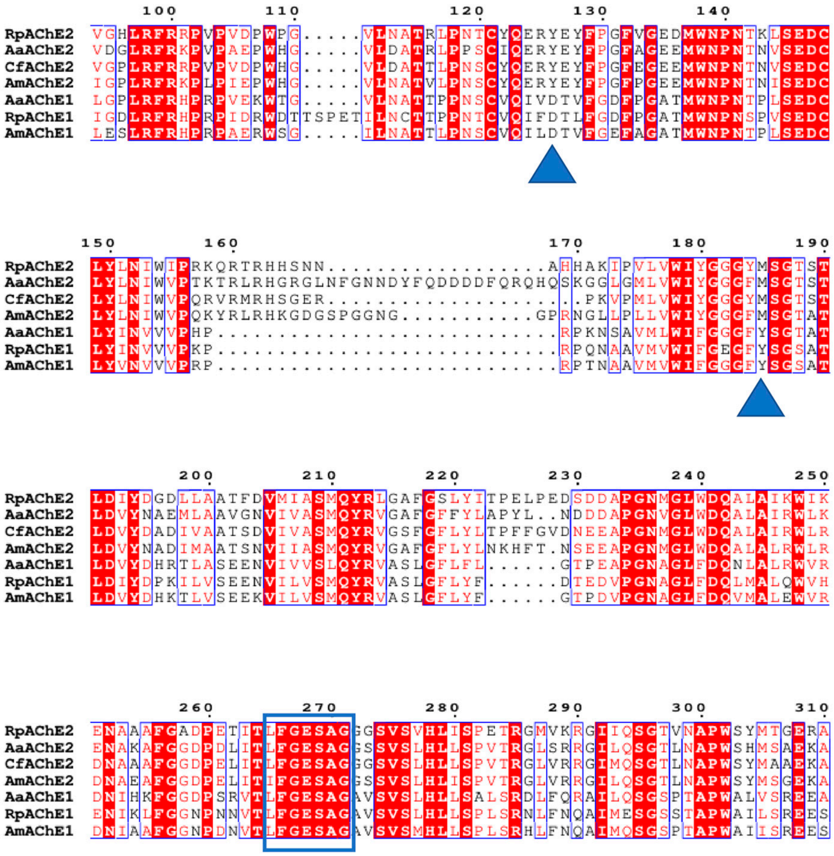

Figure S2. Alignment of the deduced amino acid sequences encoded by CfAChE2 *Coptotermes formosanus* and orthologs from other insect species including *Apis mellifera ligustica* , *Aedes albopictus*, and *Rhopalosiphum padi*. The Y/D and M/Y typing sites distinguishing insect AChE1 and AChE2 are indicated with blue arrowheads.The AChE signature motif is boxed. Sequence alignment was visualized using ESPript 3.2.

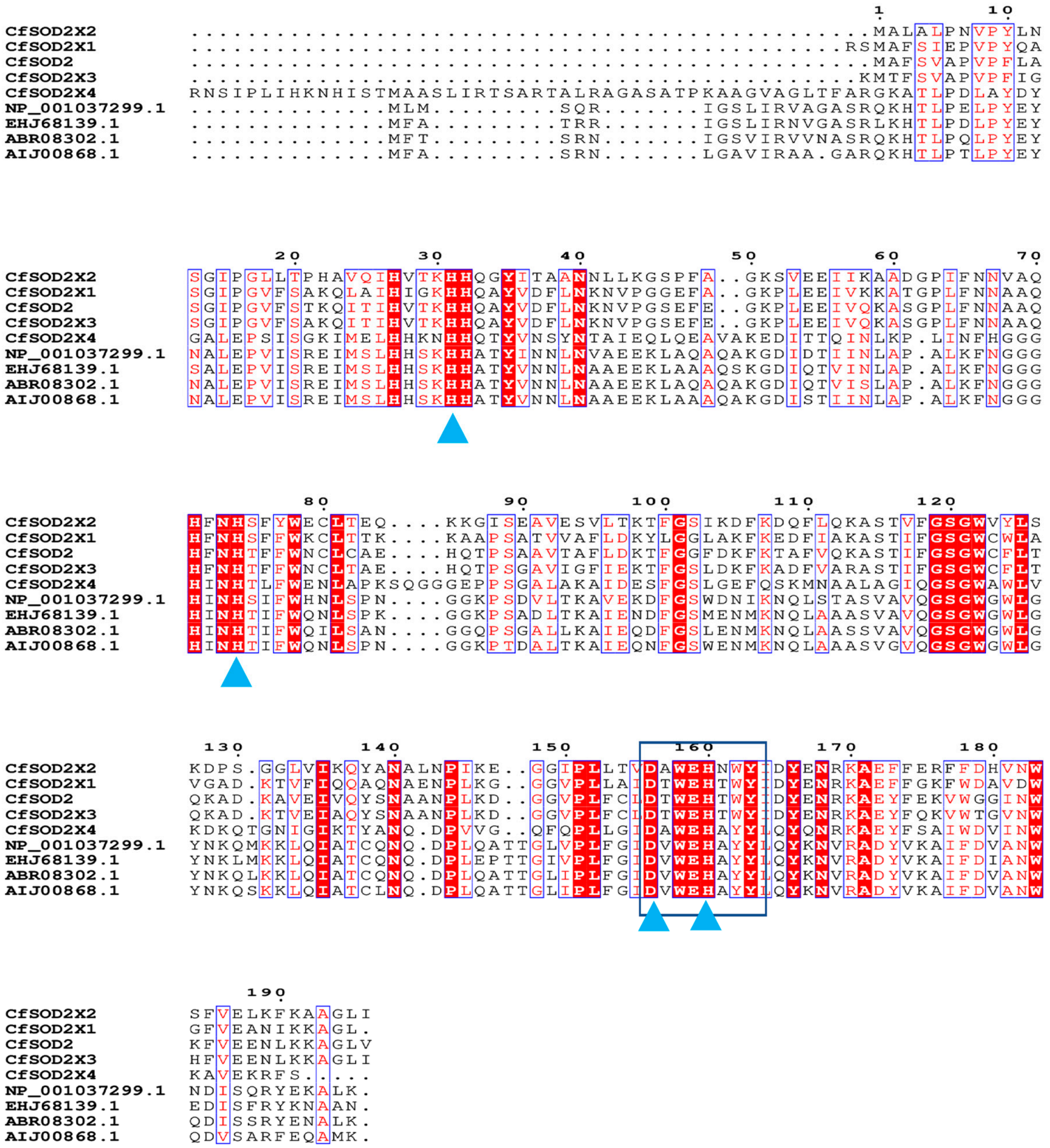

Figure S3. Alignment of the deduced amino acid sequences encoded by CfSOD2 and its four variants (CfSOD2X1, CfSOD2X2, CfSOD2X3, CfSOD2X4) from *Coptotermes formosanus* and orthologs from other insect species including *Bombyx mori* (NP_001037299.1), EHJ68139.1 (*Danaus plexippus*), ABR08302.1 (*Galleria mellonella*), and AIJ00868.1 (*Plutella xylostella*). Four conserved manganese-binding residues are indicated with blue arrowheads. The MnSOD signature motif is boxed. Sequence alignment was visualized using ESPript 3.2.
